# The Germ Cell Nuclear Proteins hnRNP G-T and RBMY Activate a Testis-Specific Exon

**DOI:** 10.1371/journal.pgen.1000707

**Published:** 2009-11-06

**Authors:** Yilei Liu, Cyril F. Bourgeois, Shaochen Pang, Marek Kudla, Natacha Dreumont, Liliane Kister, Yong-Hua Sun, James Stevenin, David J. Elliott

**Affiliations:** 1 Institute of Human Genetics, Newcastle University, Newcastle upon Tyne, United Kingdom; 2 IGBMC Department of Functional Genomics, Illkirch, France; 3 INSERM U964, Illkirch, France; 4 CNRS UMR 7104, Illkirch, France; 5 University of Strasbourg, Strasbourg, France; 6 State Key Laboratory of Freshwater Ecology and Biotechnology, Institute of Hydrobiology, Chinese Academy of Sciences, Beijing, China; 7 Department of Genetics, University of Warsaw, Poland; The University of North Carolina at Chapel Hill, United States of America

## Abstract

The human testis has almost as high a frequency of alternative splicing events as brain. While not as extensively studied as brain, a few candidate testis-specific splicing regulator proteins have been identified, including the nuclear RNA binding proteins RBMY and hnRNP G-T, which are germ cell-specific versions of the somatically expressed hnRNP G protein and are highly conserved in mammals. The splicing activator protein Tra2β is also highly expressed in the testis and physically interacts with these hnRNP G family proteins. In this study, we identified a novel testis-specific cassette exon TLE4-T within intron 6 of the human *transducing-like enhancer of split 4* (*TLE4*) gene which makes a more transcriptionally repressive TLE4 protein isoform. TLE4-T splicing is normally repressed in somatic cells because of a weak 5′ splice site and surrounding splicing-repressive intronic regions. TLE4-T RNA pulls down Tra2β and hnRNP G proteins which activate its inclusion. The germ cell-specific RBMY and hnRNP G-T proteins were more efficient in stimulating TLE4-T incorporation than somatically expressed hnRNP G protein. Tra2b bound moderately to TLE4-T RNA, but more strongly to upstream sites to potently activate an alternative 3′ splice site normally weakly selected in the testis. Co-expression of Tra2β with either hnRNP G-T or RBMY re-established the normal testis physiological splicing pattern of this exon. Although they can directly bind pre-mRNA sequences around the TLE4-T exon, RBMY and hnRNP G-T function as efficient germ cell-specific splicing co-activators of TLE4-T. Our study indicates a delicate balance between the activity of positive and negative splicing regulators combinatorially controls physiological splicing inclusion of exon TLE4-T and leads to modulation of signalling pathways in the testis. In addition, we identified a high-affinity binding site for hnRNP G-T protein, showing it is also a sequence-specific RNA binding protein.

## Author Summary

This study investigates tissue-specific alternative splicing, which plays a key role in generating diversity in animal cells. We found a new testis-specific exon in a human homologue of the important *Drosophila* developmental regulator Groucho, which is activated by germ cell RNA binding proteins. By analyzing splicing control of this exon, we elucidated how variations in the activity and expression of splicing regulators together counterbalance splicing activation, and achieve more tightly regulated physiological splicing patterns. We find that although this new human testis-specific exon is not conserved in mice, it is functionally important in that it encodes a peptide which increases the activity of this developmental regulator as a transcriptional repressor. This study provides new insights into how signalling pathways are evolving in human germ cells and the possible molecular defects that might be occurring in infertile men who have genetic deletions of germ cell-specific RNA binding proteins.

## Introduction

Alternative splicing plays a key role in expanding the coding potential of the human genome by enabling multiple mRNAs to be made from even single genes. Regulated alternative splicing is likely to be important in many if not all developmental pathways in metazoans, and has been proven to be essential in the mouse for normal cardiac, neural and thymus function [1]–[3]. Particularly high levels of alternative splicing have also been observed in the testis [4]–[7]. A relatively unique feature of the testis is that it is the site of an extensive developmental process which is maintained in the adult, and involves the coordinated division and differentiation of huge numbers of cells. An adult human testis produces 10^8^ sperm/day [8]. Alternative splicing is probably important throughout germ cell development, and is known to play a critical role in transcriptional re-programming after meiosis where it converts the transcription factor cAMP responsive element modulator from an antagonist to a potent activator required for transcription from an array of promoters in round spermatids [9],[10].

The reasons for high levels of alternative splicing in the testis are unknown, but might indicate a particular requirement for increased transcript isoforms in this tissue. There is an increased frequency of species-specific splicing events in the testis (not conserved between mouse and human) compared with that detected in the brain [11]. This might indicate “extra noise”, although an increase in alternative splicing might be itself one of the mechanisms driving the rapid evolution of reproductive systems between species including between mice and humans [12]. Consistent with this idea, in general gene expression control is rapidly evolving in the germline, with even some entire genes encoding regulatory components being entirely missing in mouse and yet essential in human and *vice versa* [13],[14].

Alternative patterns of pre-mRNA splicing in different cell types and tissues are in part controlled by cellular modulations in the concentration of nuclear RNA binding proteins [15],[16]. Individual pre-mRNAs are thought to respond differently to the cellular concentrations of nuclear RNA binding proteins depending on their sequence content (or splicing code). Consistent with the observed high frequency of alternative splicing in the testis, distinct patterns of splicing regulators are expressed during spermatogenesis [11]. Two important RNA binding proteins which are only expressed in the male germline during spermatogenesis are RBMY (RNA-binding motif gene on Y chromosome) and hnRNP G-T. *RBMY* genes are conserved on all mammalian Y chromosomes, and are homologous to the X-chromosome gene *RBMX* which encodes hnRNP G protein [13],[14]. *HNRNPGT*, a retrotransposed copy of *RBMX*, is conserved in all placental mammals, although another gene more recently retrotransposed from *RBMX* is already starting to degenerate in the rodent lineage [15]. *RBMY* is a candidate gene for causing the meiotic arrest observed in men with Y chromosome deletions in the AZFb region [16] and haploinsufficiency of hnRNP G-T protein prevents functional spermatogenesis in the mouse [15]. These genetic data and evolutionary conservation are indicative of an important function, but to date there are no known physiological target transcripts for either hnRNP G-T or RBMY proteins in the testis. RBMY and hnRNP G-T proteins each interact with a network of RNA binding proteins which regulate splicing. These interacting proteins belong to the SR- and SR-related families of splicing regulators (SRp20 and Tra2β) and STAR (signal transduction and activation of RNA) family of proteins (Sam68 and T-STAR) [17]–[20], but the functional consequences of these protein interactions on the selection of alternative splice sites within the testis are not known.

To search for candidate transcripts which might be regulated in human germ cells by RBMY and hnRNP G-T we carried out an EST-based computer analysis and identified a novel testis-specific exon in the functionally important *TLE4* gene. We find the splicing pattern of TLE4-T is established through a combinatorial control mechanism between the Tra2β and RBMY/hnRNP G-T splicing regulator proteins.

## Results

### Identification of a testis-enriched spliced form of the TLE4 mRNA in humans

To identify candidate alternative splicing events which might be regulated by the splicing code in germ cells, 2954 human cassette exons expressed in the testis were retrieved from the HOLLYWOOD alternative splicing database [21]. A few stringent screening steps were taken to enable the identification of exons exclusively expressed in the testis (Figure S1). First, we presumed that the annotated Ensembl transcripts represented the major transcription isoform. All the exons that matched their Ensembl transcripts (version 38) were removed. The remaining 666 exons were blasted against NCBI human EST (expressed sequence tag) database, and the tissue origin of each EST hit was checked. All exons with EST coverage from tissues other than testis were removed. This resulted in a collection of 150 putative testis-specific exons. The last step was a manual check to eliminate any exons with splicing ambiguities or located in UTRs (untranslated regions). Finally a list of 102 putative testis-specific exons was compiled (Table S1). The majority of these exons were out-of-frame and not conserved in mouse, which is consistent with the observations from other studies which indicate that testis is a tissue enriched in species-specific alternative splicing events [11].

**Figure 1:**
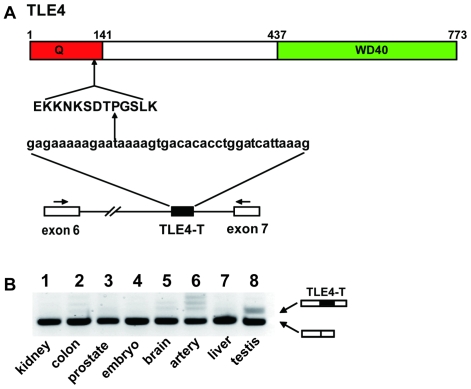
An alternative exon in the TLE4 gene is specifically spliced in the human testis. (A) Cartoon of the *TLE4* gene protein showing the N-terminal Glutamine rich (Q) domain and the C-terminal WD40 domain. A testis-specific exon between exons 6 and 7 of the human *TLE4* gene inserts an extra 39 nucleotides encoding 13 amino acids into the TLE4 protein. (B) Tissue distribution of the TLE4-T exon assayed in a panel of RNAs made from human tissues. The positions of the primers used for RT–PCR are shown as arrows above exons 6 and 7. On the agarose gel, the upper band corresponds to the product including the TLE4-T exon, while the lower band corresponds to the RT–PCR product resulting from direct splicing between exons 6 and 7. The TLE4-T splice isoform is specifically enriched in the testis.

One testis-specific exon identified in this search was a cassette exon within intron 6 of the *TLE4* gene (*Transducin Like Enhancer of split 4*). *TLE4* is a human homologue of the *Drosophila* gene *Groucho*. The TLE4-T exon architecture comprises a strong 3′ splice site (score 9.2) and a weak 5′ splice site (score 4.9) based on the Splice Site Score Calculation program (http://rulai.cshl.edu/new_alt_exon_db2/HTML/score.html). When spliced into the TLE4 mRNA, exon TLE4-T encodes an extra 13 amino acids which are inserted in frame into the C-terminal proximal region of the Q domain (a glutamine rich domain which mediates homo/hetero-dimerization of the TLE4 protein) [22]–[24] (Figure 1A). Confirming our bioinformatic approach based on the HOLLYWOOD-RNA Alternative Splicing data base, RT–PCR analysis using RNAs from different human tissues indicated that TLE4-T is predominantly spliced in the testis (Figure 1B).

### Splicing inclusion of TLE4-T creates a more strongly repressive TLE4 isoform in the human testis

**Figure 2:**
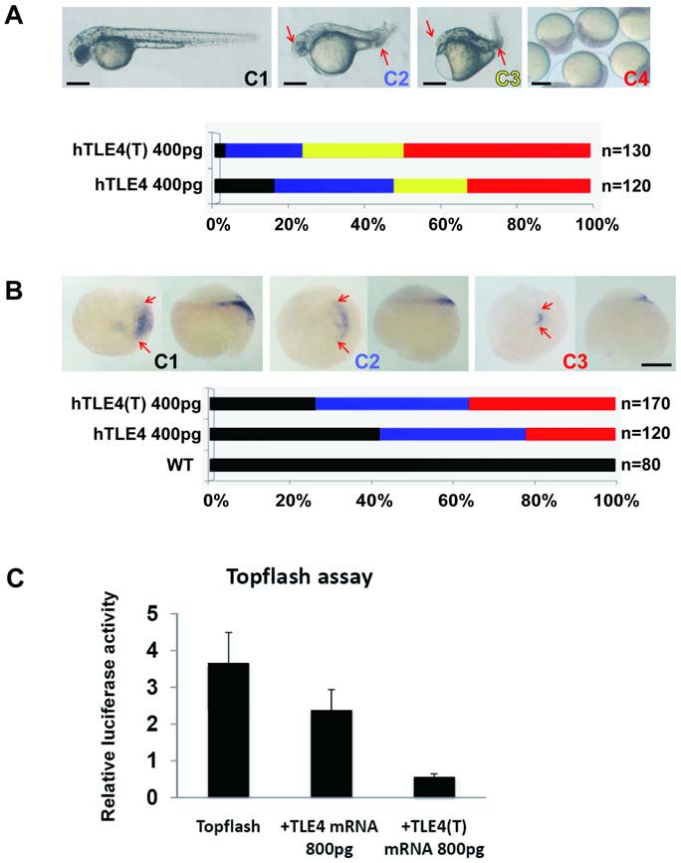
TLE4-T exon modulates the activity of the cognate TLE4 protein. (A) Over-expression of the human TLE4(T) mRNA isoform has a stronger ventralising effect than the ubiquitously expressed TLE4 mRNA on early zebrafish development. A spectrum of phenotypes was observed ranging from normal to very strongly ventralised. Representative embryos in each class of embryo are shown. The percentage of different phenotypes observed in the zebrafish embryos after mRNA injection are shown as a bar chart, with colours black, blue, yellow and red corresponding to the proportion of phenotypes C1–C4 respectively. C1 represents normal development, C2 represents weak ventralisation (smaller eyes and enlarged blood island are pointed out by red arrows), C3 represents strong ventralisation (as shown by loss of eyes and hugely enlarged blood island, red arrows), C4 represents early developmental arrest at blastula stage. Embryos of C1, C2, and C3 are shown in lateral view with anterior to the left and dorsal to the top. (B) Injection of the human TLE4-T mRNA isoform into zebrafish embryos has a stronger repressive effect on expression of the Wnt/β-catenin target gene *dharma* than injection of the TLE4 mRNA isoform. Embryos representing the different *dharma* expression patterns C1–C3 are shown. The percentage of different blastocyst *dharma* expression phenotypes is shown as a bar chart, with colours black, blue and red corresponding to the proportion of phenotypes C1–C3 respectively. Embryos are shown in lateral view with animal pole to the top and dorsal to the right. Scale bars represent 250 µm in (A) and (B). (C) TOPFLASH assay in zebrafish embryos. Relative luciferase activity represents the relative Wnt/β-catenin signalling activity in zebrafish embryos receiving different mRNAs. After injection of 100 pg Topflash reporter and 10 pg Renilla reporter, either 800 pg TLE4 mRNA or 800 pg TLE4(T) mRNA was injected into each embryo. The vertical bar represents mean relative luciferase activity ± SD.

Groucho/TLE family proteins are transcriptional co-repressors required in many developmental processes including pattern formation, segmentation, sex determination, and neurogenesis [24],[25]. One major function of Groucho/TLE proteins is to repress Wnt/β-catenin transcription activity by binding to TCF proteins [26],[27], but Groucho/TLE proteins also repress transcription of genes activated by the Notch and Hedgehog signaling pathways. Although Groucho/TLE family proteins are well conserved in most metazoans, the TLE4-T exon is absent in the mouse and any other more distant lineage to humans. This suggests that exon TLE4-T does not play a conserved role in TLE4 mediated functions, but might modulate the function of the TLE4 protein in human germ cells. To test if inclusion of the peptide encoded by TLE4-T has any influence on the protein activity of human TLE4, we carried out an *in vivo* activity assay in zebrafish embryos by ectopic over-expression of human TLE4 proteins. Zebrafish has four Groucho/TLE homologues (2 homologs to human *TLE2* and 2 homologues to human *TLE3*) which share over 80% identity with human *TLE4* [28]. Consistent with its role in the Wnt/β-catenin pathway and the significance of maternal Wnt/β-catenin activity on the development of dorsal axis [29], over-expression of *Groucho* affects early dorsal-ventral pattern formation in zebrafish and represses transcription of maternal Wnt/β-catenin target genes including *dharma* [30] (our unpublished data). As expected, microinjection of the somatic form of human TLE4 mRNA (hTLE4) into early zebrafish embryos had a ventralising effect on early zebrafish development, indicating functional conservation of TLE/Groucho proteins between zebrafish and human. After injection of 400 pg hTLE4 mRNA per embryo, around 50% of the zebrafish embryos were either normal (phenotype C1 in Figure 2A) or weakly ventralised (phenotype C2 in Figure 2A). Injection of the same dose of the testis-specific isoform of human TLE4 mRNA, hTLE4(T) which contains exon TLE4-T, resulted in a more strongly ventralised effect, with only around 25% of embryos being normal or weakly ventralised and the remainder being more severely affected (phenotypes C3 and C4 in Figure 2A).

Similar results were obtained by analysing expression of the Wnt/β-catenin target gene *dharma* in zebrafish blastulas. Non-injected blastulas showed strong staining of *dharma* expression at their dorsal margin (phenotype C1 in Figure 2B). Ectopic expression of the hTLE4(T) isoform resulted in a stronger effect on the repression of *dharma* transcription (more embryos showing phenotypes C2–3 and less embryos showing the wild type phenotype C1) compared with ectopic expression of the constitutive isoform hTLE4 (Figure 2B).

To quantitatively assay the level of β-catenin signalling in the presence of hTLE4 or hTLE4(T), we measured the activity of the β-catenin responsive reporter Topflash [31] following mRNA injection into zebrafish embryos. Although hTLE4 and hTLE4(T) both repressed the Topflash reporter (p<0.05 for both conditions), hTLE4(T) showed higher repressive activity than hTLE4 (p<0.05) (Figure 2C). These experiments indicated that the hTLE4(T) protein isoform has an enhanced Wnt/β-catenin repressive activity compared with hTLE4, and so might also show increased transcriptional repressive activity in this and other signaling pathways regulated by TLE4 protein in human germ cells.

### The TLE4-T exon is normally repressed through intronic elements

**Figure 3:**
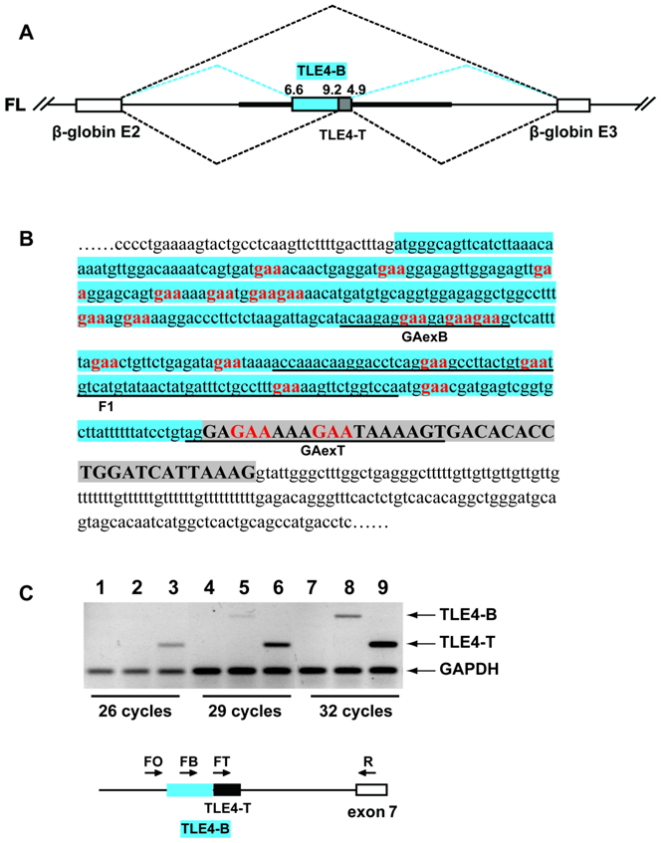
Structure of the minigene designed to analyse TLE4 pre–mRNA splicing. (A) Cartoon of minigene in which exon TLE4-T and the flanking introns were cloned into pXJ41 to give the FL minigene. The exons are shown as boxes, and the introns as lines. The TLE4 flanking intron fragments are shown as a thicker line. mRNAs composed of three different combinations of exons were made from this minigene. The β-globin exons could be directly spliced together (upper broken line), with TLE4-T exon spliced in between (bottom broken line), or with a longer TLE4 alternative exon called TLE4-B spliced into the minigene encoded mRNA (middle blue broken line). (B) The nucleotide sequence of the TLE4-T exon (highlighted grey) and the TLE4-B exon (specific TLE4-B region highlighted blue; notice that the TLE4-B exon also uses the same 5′ splice site as TLE4-T). The GAA motifs are in bold red, although there are further GA-rich motifs that could be additional Tra2β binding sites. The immediately flanking intron sequences are shown in lower case unshaded. The TLE4-B exon contains stop codons which would truncate the TLE4 reading frame, while the TLE4-T exon maintains the open reading frame. Probes used for UV crosslinking experiments are underlined. (C) RT–PCR experiment on endogenous human testis RNA showing the TLE4-B transcript is expressed at much lower level than the TLE4-T transcript in the human testis. Three PCR reactions using one of the forward primers FO, FB, FT, together with the reverse primer R were performed for 3 different cycle numbers, quantitatively showing the abundance of transcripts containing TLE4-B (lanes 2, 5, 8) and TLE4-T (lanes 3, 6, 9) relative to *GAPDH*. The lower panel shows the position of the primers relative to the exons assayed. Lanes 1, 4, 7 are negative control using primer FO which is located immediately upstream of B exon 3′ splice site.

**Figure 4:**
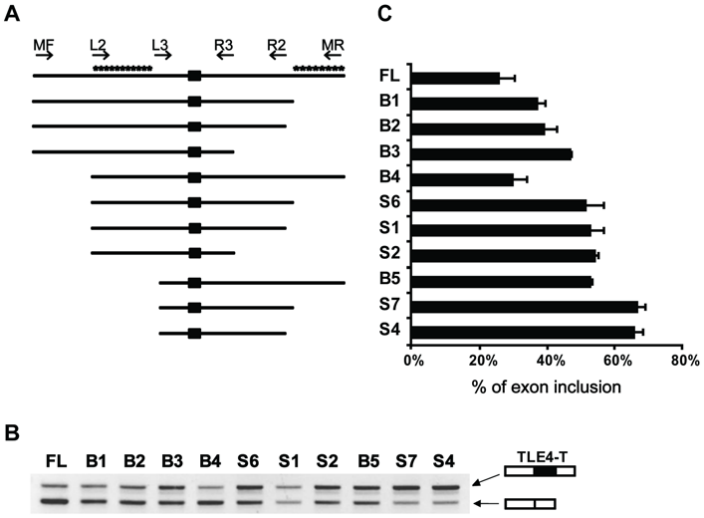
Splicing of the TLE4-T exon is repressed in somatic cells. (A) Schematic diagrams show the structures of the full length minigene TLE4-T (FL) and 10 small minigenes (B1-S4) with partially truncated introns (drawn to scale). The primers used to clone these minigenes are shown on top of the minigenes except, due to limited space, for primer R5 which is almost coincident with R2. R5 was used as the reverse primer of B1, S6 and S7. The putative intronic repressive elements are labelled by asterisks. (B) RT–PCR analysis showing the splicing pattern of each of the minigenes in HEK293 cells. (C) Histogram showing the average TLE4-T exon inclusions from 3 independent sets of RT–PCR experiments.

TLE4-T is normally absent in somatic tissues but is included in the human testis (Figure 1B). In order to dissect splicing regulation of the TLE4-T exon, we constructed a minigene containing the exon TLE4-T together with around 600 bp of both flanking intron sequences cloned between β-globin exons (Figure 3A). Pre-mRNAs from this full length (abbreviated FL) minigene efficiently recapitulated the splicing pattern of the endogenous *TLE4* gene by largely skipping exon TLE4-T in HEK293 cells (Figure 4B and 4C, FL). We made a series of minigenes with different intron lengths (Figure 4A), and found that TLE4-T splicing inclusion was progressively activated by gradually taking out flanking intron sequence. The mRNAs containing exon TLE4-T (the upper band in the gel shown in Figure 4B) changed from the minor isoform for the full length (FL) minigene, to the major isoform in minigenes S7 and S4 (Figure 4B and 4C). In particular the deletion of two flanking intron regions between primers L2 and L3 in the upstream intron and between primers R2 and MR in the downstream intron resulted in very strong splicing activation of TLE4-T.

### Identification of nuclear proteins that bind to the TLE4-T exon

**Figure 5:**
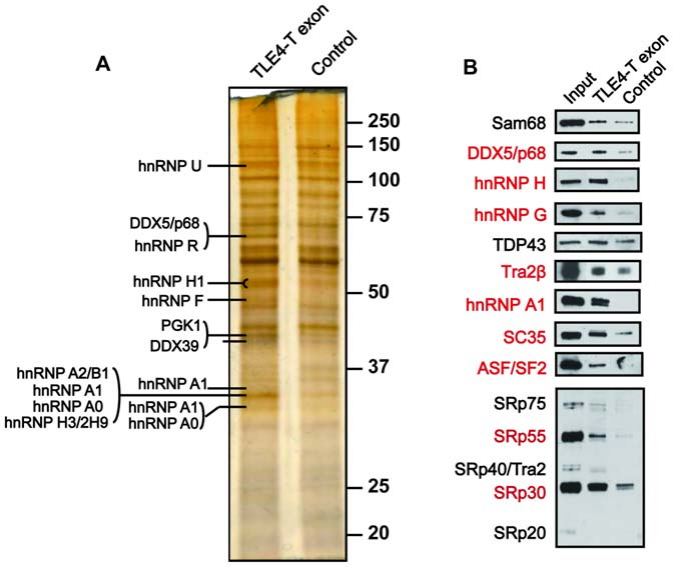
Analysis of the proteins which bind to the TLE4-T exon. Transcribed RNAs corresponding to the TLE4-T exon and the negative control (GST-89) were immobilised on beads and incubated in nuclear extracts made from untransfected HeLa cells. (A) Silver stained gel of proteins bound to the two RNA sequences. Strong protein bands that differentially bound to TLE4-T and the control RNA were identified by mass spectroscopy and are labelled with protein names. (B) Pull down samples were analysed by Western blotting with antibodies specific for splicing factors. Proteins labelled in red showed more significant binding to the TLE4-T exon than to the control RNA sequence.

The above results indicate that splicing of exon TLE4-T is normally repressed by flanking intronic elements which prevent its inclusion in HEK293 cells, and suggest a mechanism exists to counteract this repression within the testis. To next test the role of the exonic sequences in splicing control, we carried out a pull down assay to identify nuclear RNA binding proteins which bind to and might regulate TLE4-T splicing. Exon TLE4-T and a control RNA [18] were covalently attached to agarose beads and incubated in HeLa cell nuclear extract. Proteins bound to agarose beads were identified by PAGE and silver staining/mass spectrometry and/or Western blot (Figure 5). A number of proteins were detected as bound to the TLE4-T exon, but not to the control RNA. These proteins included known splicing activators such as members of the SR (serine/arginine-rich) protein family: SRp30 (both SC35 and ASF/SF2), SRp55, and the SR-related protein Tra2β (in agreement with the presence of a purine rich element in the TLE-T exon); Sam68 (weak binding compared with the control RNA); hnRNP proteins (hnRNP H, hnRNP A1 and hnRNP G) and the RNA helicase p68. Co-expression of each of the SR proteins with the TLE4 FL minigene showed that none of these activated splicing inclusion of TLE4-T (Figure S2), although hnRNP A1 had a slight repressive effect (Figure S3).

### The hnRNP G-T and RBMY proteins function as co-activators of TLE4-T splicing inclusion

**Figure 6:**
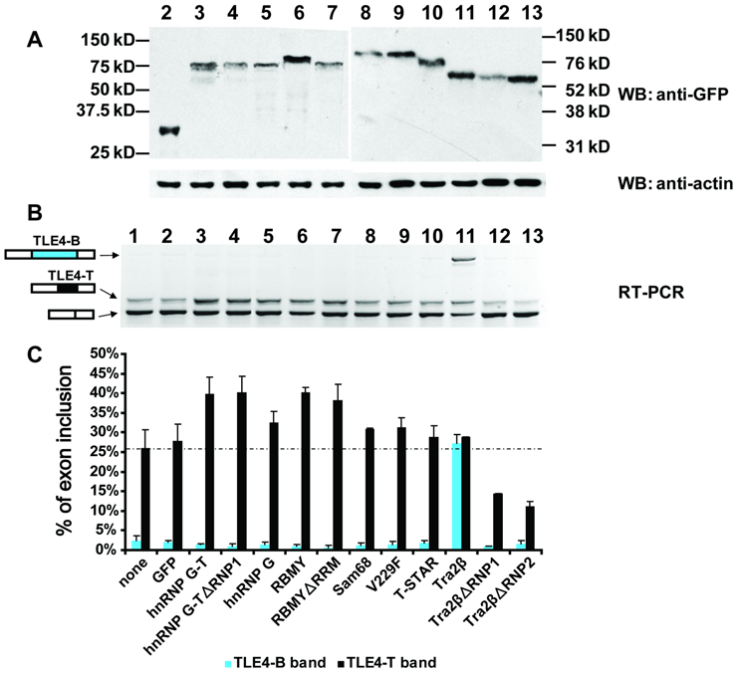
Splicing of TLE4-T is up-regulated by hnRNP G family proteins, Tra2β, and STAR family proteins. Western blot (A), RT–PCR electrophoresis (B) and densitometry (C) analysis of HEK293 cells transfected with the TLE4 FL minigene by itself, or the FL minigene cotransfected with GFP or different splicing factors fused to GFP. The same samples in different panels are lined up, and the cotransfected splicing factors are indicated at the bottom of panel (C). (A) Western blot of transfected cells showing expression levels of each of the transfected, epitope tagged proteins compared to endogenous actin protein. The amounts of transfected DNA were adjusted to give similar levels of protein expression over multiple replicate experiments. (B) Analysis of TLE4 minigene splicing patterns assayed by RT–PCR and agarose gel electrophoresis. (C) Bar chart showing the densitometric analysis of the percentage of the two alternative splice isoforms. The broken line in the bar chart represents the average level of TLE4-T splicing detected when the minigene was transfected by itself into HEK293 cells.

The observed binding of Tra2β and hnRNP G to TLE4-T was of particular interest in relation to the splicing inclusion of this exon in the testis. Tra2β is known to activate the testis-specific HIPK3-T exon and is over-expressed in the testis [18], and the hnRNP G homologous proteins RBMY and hnRNP G-T have been implicated in alternative splicing decisions in the testis but have no known pre-mRNA targets. In order to see if these proteins could affect splicing of exon TLE4-T we carried out co-transfection experiments with the FL minigene. Co-transfection of hnRNP G with the TLE4-T minigene led to moderate splicing activation of TLE4-T (Figure 6B, lane 5) but exon TLE4-T was even more efficiently spliced in response to hnRNP G-T and RBMY (Figure 6B, compare lanes 3 and 6 to 5). Splicing activation of TLE4-T by RBMY and hnRNP G-T was RRM-independent (Figure 6B, lanes 4 and 7). This indicates that RBMY and hnRNP G-T proteins are functioning as splicing coregulators to activate TLE4-T splicing rather than through direct contact with RNA. To monitor expression efficiencies within and between each experiment, protein samples were prepared in parallel from transfected cells and probed by Western blots using antisera to GFP (green fluorescent protein, to detect the GFP tag on the transfected splicing regulators) and actin (Figure 6A).

### Although it functions as a splicing co-activator of TLE4-T, the hnRNP G-T protein is a sequence-specific RNA binding protein

**Figure 7:**
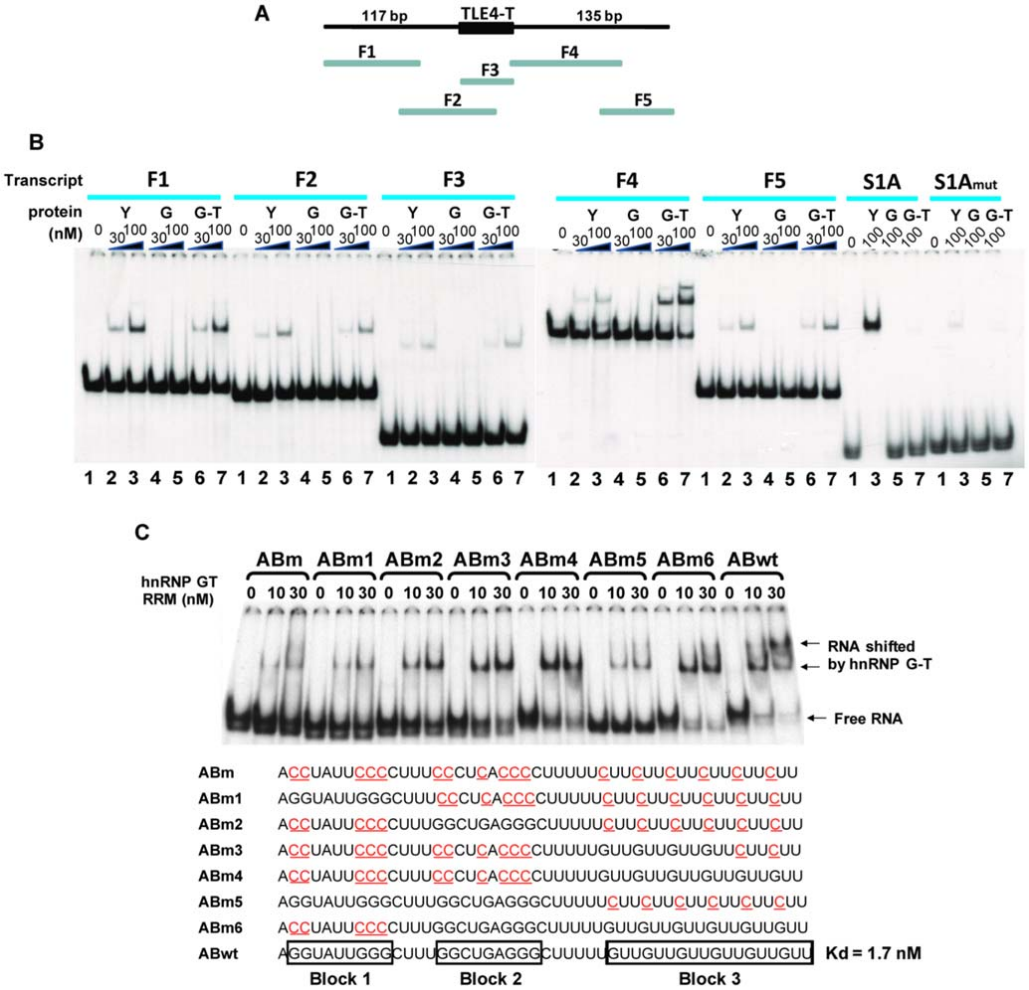
Identification and dissection of a direct binding site for hnRNP G-T downstream of TLE4-T. (A) Cartoon of the TLE4-T exon and flanking introns showing the position of each of the *in vitro* transcribed RNAs F1 to F5 which were used in the EMSA experiments. (B) Mapping of interactions of RBMY, hnRNP G, and hnRNP G-T with each of the fragments F1 to F5 using EMSA. In each binding reaction, there is either no protein (lane 1) or the RRM of RBMY (Y) (30 ng at lane 2, 100 ng at lane 3), hnRNP G (G) (30 ng at lane 4, 100 ng at lane 5), and hnRNP G-T (G-T) (30 ng at lane 6, 100 ng at lane 7) was added. The positive control for RBMY protein-RNA binding is *in vitro* transcribed S1A RNA. The negative control for RBMY-RNA binding is S1Amut, in which the RBMY binding site is mutated. (C) High resolution mapping and dissection of the hnRNP G-T protein binding site identified in the *in vitro* transcribed RNA F4. The sequences of each of the *in vitro* transcribed RNAs are shown underneath a representative gel showing a complete set of interactions measured by EMSA.

The above experiments showed that RBMY and hnRNP G-T efficiently induce TLE4-T splicing as splicing co-activators, but hnRNP G physically interacted with TLE4-T RNA in pull down assays. To analyse whether these proteins can directly bind to the TLE4 pre-mRNA or not we subcloned the TLE4-T exon and surrounding intron sequence into 5 partially overlapping clones in pBluescript, transcribed these clones *in vitro* and tested the binding of each of the transcripts by EMSA (Electrophoretic Mobility Shift Assay) using recombinant RRMs from RBMY, hnRNP G-T and hnRNP G (Figure 7A and 7B). As a positive control we used the S1A sequence which we had previously shown to bind at high affinity to the RRM of RBMY [32]: as expected S1A RNA was efficiently shifted by RBMY protein but not by the other proteins (Figure 7B).

In EMSAs, hnRNP G did not directly bind any of these *in vitro* transcribed RNAs (Figure 7B, lanes 4 and 5 for each RNA), suggesting that in the pulldown experiments hnRNP G may interact indirectly with the TLE4-T exon in HeLa nuclear extracts. While it did not bind strongly to the TLE4-T exon, the RRM of RBMY interacted with fragment F1 (lanes 2 and 3). This binding was measured to have a Kd (dissociation constant) of 3.3 nM+/−0.11 (based on 2 independent assays), although all 5 RNA fragments had a much weaker affinity for RBMY compared with its SELEX winner sequence S1A. HnRNP G-T also interacted with fragment F1 (lane 6 and 7) showing a Kd of 3.9 nM+/−0.69 (based on 3 independent assays).

Surprisingly however, hnRNP G-T very efficiently shifted fragment F4 indicating a potential strong binding site for hnRNP G-T within this fragment (Figure 7B, lanes 6 and 7). Initial rough mapping (data not shown) narrowed down a strong hnRNP G-T binding site to a 47 nt sequence (ABwt) in the first half of fragment F4 with a Kd of 1.7 nM+/−0.19 (based on 3 independent assays). This 47 nt sequence consists of 3 blocks of G-rich sequence separated by some U-rich stretches (Figure 7C). Gel-shift assays (data not shown) indicated that the RRM of hnRNP G-T had an increased binding efficiency to G-rich RNA. Consistent with this the replacement of G to C (to create mutant RNA ABm) resulted in a complete loss of binding compared to the shift of wild type sequence (ABwt) (Figure 7C).

To finely map the hnRNP G-T protein-RNA interaction site, we reconverted individual blocks in the mutated transcript ABm back into G residues to create plasmids ABm1-ABm6. Comparison of the binding efficiencies of transcripts made from each of these plasmids show that block 3 has the highest affinity to the RRM of hnRNP G-T, though block 2 also showed weak binding. A comparison of the hnRNP G-T binding of transcripts made from plasmids ABm4 and ABm3 indicated that the more GUU repeats the RNA contains, the more efficiently hnRNP G-T protein binds to the RNA.

### Tra2β weakly activates TLE4-T splicing but very strongly activates inclusion of a variant exon using a weaker upstream 3′ splice site

We similarly investigated splicing regulation of TLE4-T by Tra2β (Figure 6). Co-transfection of Tra2β weakly activated splicing of TLE4-T but very potently induced splicing inclusion of a further TLE4 splicing isoform we annotated as TLE4-B (Figure 6B, lane 11). Subcloning and sequencing revealed this alternative B exon uses the same 5′ splice site as TLE4-T but a weak alternative 3′ splice site (score: 6.6) located 339 bp upstream (Figure 3A and 3B). Since the splicing of this TLE4-B exon was not initially identified in the testis or other tissues with primers on flanking constitutive exons, we performed another RT–PCR using cassette exon specific primers to assess the abundance of the TLE4-B exon containing isoform. Although physiologically detectable, the TLE4-B exon isoform was expressed at a much lower level in the testis compared to the TLE4-T exon isoform (Figure 3C, compare lanes 5 with 6 and lanes 8 with 9).

Unlike for hnRNP G-T or RBMY, splicing activation of TLE4-T and TLE4-B by Tra2β was dependent on the RRM sequence of Tra2β. The repressive effect of Tra2β ΔRNP1 or ΔRNP2 proteins on TLE4-T splicing may be due to a dominant-negative effect. Tra2β can interact with itself and other splicing factors like hnRNP G. The over-expression of the Tra2β ΔRNP1 and ΔRNP2 mutants probably sequesters endogenous Tra2β and other splicing regulators such as hnRNP G which might be essential for endogenous low level of splicing of the TLE4 cassette exons in HEK293 cells.

**Figure 8:**
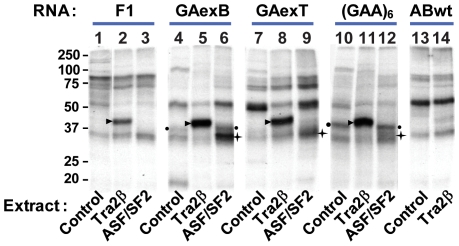
UV-crosslinking assay validating the direct binding of Tra2β to TLE4-T and TLE4-B exons. The bands showing the crosslinking of exogenous Tra2β and ASF/SF2 to the transcripts are indicated by black triangles and stars, respectively. The black dots label the RNA transcripts bound by a protein that most likely corresponds to endogenous Tra2β. No significant crosslinking to any of the RNA probes was detected in the absence of transfected Tra2β (lanes 1, 4, 7, 10 and 13), whereas a strong crosslinking was detected to a (GAA)_6_ sequence probe which is a canonical Tra2β binding site (lane 11). Moderately strong crosslinking was detected to the GA(A) repeat sequence from TLE4-T (lane 8), and very strong crosslinking to the GA(A) repeat sequence from TLE4-B (lane 5). As a control, no crosslinking was detected to the ABwt probe, which contains the binding site of hnRNP G-T from the intron downstream of TLE-T (lane 14), and very weak crosslinking to the F1 probe which has a few dispersed GA(A) repeats (lane 2). ASF/SF2 bound most strongly to GAexB, but much less efficiently to GAexT. The sequences of F1, GAexB, GAexT are underlined in Figure 3B; the ABwt sequence is given in Figure 7C.

Tra2β binds to RNA sequences containing purine-rich GAA-like motifs [18],[33], and GAA repeats are present in both the TLE4-T exon and upstream region within the TLE4-B exon. To investigate why TLE4-B was so strongly selected in response to Tra2β, we carried out a UV-crosslinking assay to measure binding of Tra2β to these sequences. This showed that the GA(A)-enriched sequences downstream of the weak TLE4-B 3′ splice site bind more strongly to Tra2β than the GA(A)-rich sequences within TLE4-T (Figure 8), comparing 5 GA/GAA motifs in GAexB to 3 GA/GAA motifs in GAexT (Figure 3B). There are further candidate GA(A) sites in the TLE4-B exon we did not directly monitor for Tra2β binding. These provide an explanation why the TLE4-B 3′ splice site is so strongly selected by Tra2β. Interestingly, the splicing activator ASF/SF2 was able to weakly bind TLE4-T (Figure 8, lane 9), but interacted strongly with the GAA-rich sequence of TLE4-B exon (Figure 8, lane 6), which is consistent with its positive effect on TLE4-B inclusion (Figure S2).

### The balance between expression levels of hnRNP G family proteins and Tra2β controls the TLE4 pre-mRNA splicing pattern

**Figure 9:**
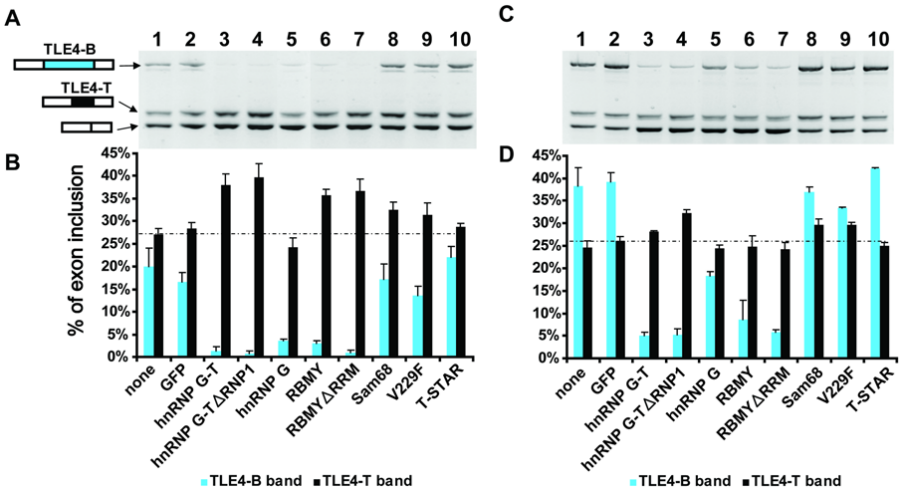
The balance of specific hnRNP G family proteins controls the splicing pattern of TLE4 pre–mRNA. RT–PCR analysis of the splicing pattern of the TLE4-T minigene from cells cotransfected with different splicing factors (indicated at the bottom of panel B or D) along with a constant amount of Tra2β (500 ng) (A) or with an increased constant amount of Tra2β (1 µg) (C) respectively. (B) and (D) Bar chart showing quantitation data. The same samples in different panels are lined up. The broken line in the bar chart represents the average level of TLE4-T splicing detected when the minigene was transfected by itself into HEK293 cells.

Since Tra2β expression is up-regulated in the testis [18], this raised the question of why the Tra2β-activated TLE4-B exon is detected at such low levels in this tissue. One possibility is that the TLE4-B exon will introduce stop codons into and so destabilise the TLE4 mRNA due to nonsense-mediated mRNA decay. Alternatively the expression of RBMY and hnRNP G-T proteins which interact with Tra2β might shift splicing of the pre-mRNA away from the TLE4-B isoform. To next assess these possibilities we used the TLE4-T FL minigene to analyse the effect of co-expressing hnRNP G family proteins (i.e. hnRNP G, hnRNP G-T or RBMY) and Tra2β on the selection of both alternative 3′ splice sites in the TLE4 pre-mRNA (Figure 9). Expression of the FL minigene with empty GFP vector did not affect Tra2β's activation of TLE4-B exon splicing (Figure 9A and 9B, lane 2). However Tra2β-mediated splicing activation of exon TLE4-B was potently inhibited by co-expression of GFP fusion constructs containing either RBMY, hnRNP G-T or hnRNP G (Figure 9A, lanes 3–7). While each of the hnRNP G family proteins inhibited Tra2β-mediated TLE4-B splicing activation, only co-expression of hnRNP G-T and RBMY also led to efficient activation of TLE4-T exon splicing (Figure 9A and 9B, lanes 3–7).

The above experiments indicate that splicing activation of TLE4-B by Tra2β is efficiently repressed by hnRNP G family proteins. In order to test reciprocally whether RBMY and hnRNP G-T mediated splicing activation of TLE4-T could be antagonised by higher cellular concentrations of Tra2β protein we carried out similar experiments, but this time transfecting an increased dose of Tra2β (Figure 9C and 9D). We found that transfection of cells with an increased quantity of Tra2β did indeed repress TLE4-T splicing activation by RBMY and hnRNP G-T proteins. Although we observed consistent repression of TLE4-T splicing at these increased concentrations of Tra2β protein, the normally germ cell-restricted hnRNP G-T protein was the only hnRNP G family protein which was still able to activate TLE4-T exon splicing. We also observed at these higher levels of Tra2β that hnRNP G protein was noticeably much less potent at repressing the splicing activation of TLE4-B by Tra2β than either RBMY or hnRNP G-T. As above, in each experiment the levels of transfected protein were monitored in each replicate experiment by Western blotting, and transfections adjusted to ensure equal levels of each splicing regulator protein were expressed in transfected cells (data not shown).

### T-STAR and Sam68 activate splicing of TLE4-T but do not antagonise the activity of Tra2β

The testis-specific T-STAR protein and its ubiquitously expressed homologue Sam68 also weakly activated splicing of exon TLE4-T (Figure 6A and 6B, lanes 8 and 10). Although Sam68 was detected as an interacting protein of the TLE4-T exon in pull down assays, the observed weak activation of TLE4-T splicing is most likely to depend on protein-protein interactions rather than being through direct RNA binding since the RNA binding deficient V229F mutant of Sam68 was equally able to stimulate TLE4-T splicing inclusion (Figure 6A and 6B, lane 9).

Both T-STAR and Sam68 interact with RBMY and hnRNP G-T, but not Tra2β [17] so should not inhibit the splicing activity of Tra2β if the previously observed antagonism is due to sequestration through protein-protein interactions. To test this, we carried out similar co-transfections of STAR proteins with Tra2β. Consistent with predictions, unlike the hnRNP G family proteins, neither T-STAR nor Sam68 inhibited the ability of Tra2β to stimulate splicing of TLE4-B (Figure 9, lane 8–10). This suggests a model in which the known direct protein interactions between RBMY and hnRNP G-T with Tra2β [17],[34] are essential to antagonise selection of the TLE4-B 3′ splice site by Tra2β through protein sequestration.

## Discussion

**Figure 10:**
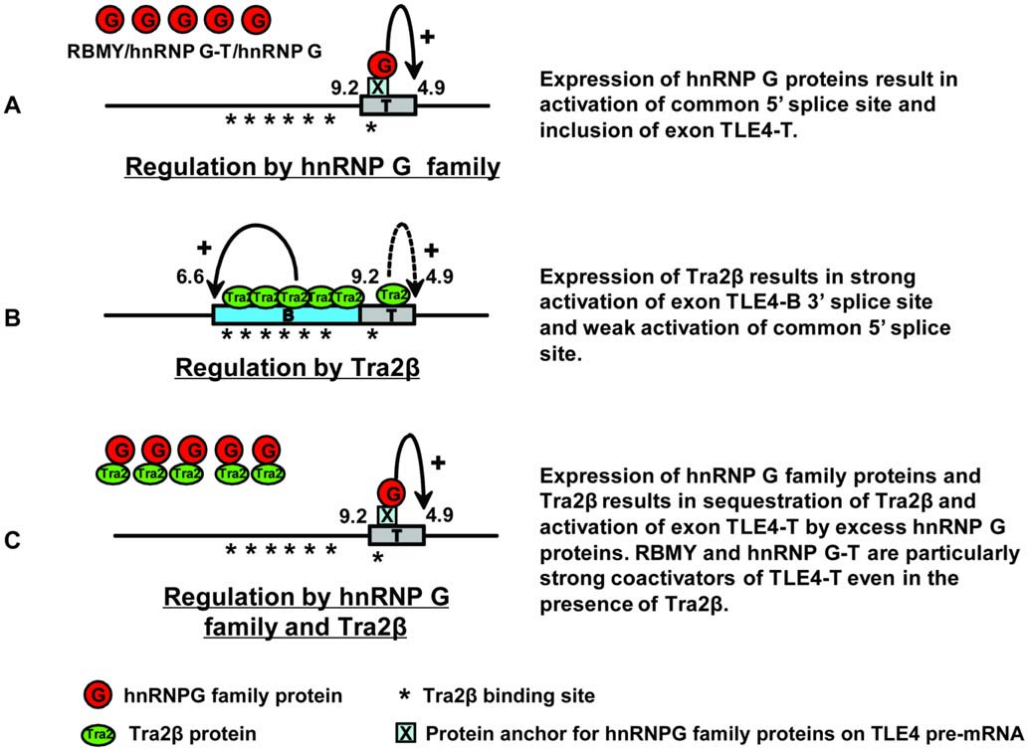
Model showing the splicing regulation of TLE4-T by hnRNP G proteins and Tra2β. (A) High levels of hnRNP G family protein expression lead to splicing activation of the TLE4-T exon. The hnRNP G proteins do not directly bind to RNA, but activate splicing indirectly through an RRM-independent mechanism. Here a bridging protein is shown as X. (B) High levels of Tra2β lead to strong activation of the TLE4-B 3′ splice site. As a consequence of the increased nuclear concentration of Tra2β protein, the multiple GAA-rich binding sites in the TLE4 pre-mRNA are occupied by Tra2β protein. We have directly mapped one of the TLE4-B and the TLE4-T Tra2β-binding site by crosslinking. (C) If both Tra2β and hnRNP G family proteins are expressed, these two sets of proteins will mutually antagonise each other. Any surplus hnRNP G protein or Tra2β will be then able to activate splicing of TLE4-T. In this case the hnRNP G family proteins are shown activating splicing of this exon.

In this study we have identified a testis-specific cassette exon TLE4-T within the pre-mRNA of an important transcriptional repressor. TLE4-T splicing is primarily repressed in somatic cells through a combination of a weak 5′ splice site and the action of surrounding intronic silencing elements. Although normally tightly repressed in somatic cells, TLE4-T exon splicing can be activated through ectopic expression of Tra2β or members of the hnRNP G family amongst the panel of proteins we identified as bound to this exon through pulldown analyses (summarised in Figure 10A and 10B). Immunoprecipitation and yeast 2 hybrid experiments have shown Tra2β and hnRNP G family proteins directly physically interact [17], and can either mutually antagonise each other through protein sequestration or competitive RNA binding (Dreumont et al, in preparation) [34],[35] or function synergistically [36]. In *in vitro* splicing reactions, addition of regions of the RBMY protein which are able to interact with Tra2β protein blocked its ability to activate splicing of a cassette exon within an artificial tropomyosin pre-mRNA [17]. Consistent with these previous observations, we found that Tra2β-mediated splicing activation of TLE4 cassette exons is effectively silenced by co-expression of hnRNP G family proteins and *vice versa* (summarised in Figure 10C). Based on this model, when both hnRNP G family proteins and Tra2β are co-expressed in HEK293 cells, the net effect on splicing actually depends on which protein predominates overall. If both hnRNP G family proteins and Tra2β are equally expressed, then TLE4-T splicing will also be repressed through mutual antagonism. However, a slight increase in the activity of either protein over the other will result in a net increase in TLE4-T splicing activation (Figure 10C).

Our data is hence consistent with the physiological splicing levels of TLE4-T resulting from changes in the concentration and relative activity of particular nuclear RNA binding proteins in germ cells. Firstly increased levels of hnRNP G family proteins are expressed in the testis because of the exclusive germ cell expression of RBMY and hnRNP G-T proteins, and the splicing response of TLE4-T to these proteins is dose dependent (data not shown). Secondly, by normalizing ectopic expression levels we found that the ubiquitously expressed hnRNP G protein functions much less potently than the germ cell specific RBMY and hnRNP G-T proteins, both in TLE4-T splicing activation and Tra2β sequestration. Hence in somatic cells hnRNP G protein alone might not be sufficient to sequester Tra2β and to override the splicing repressive genomic environment of the TLE4-T exon. However in germ cells, higher expression levels of the more potent TLE4-T splicing activators RBMY and hnRNP G-T would lead to TLE4-T splicing. While this is the first report presenting evidence for functional differences between RBMY and hnRNP G-T proteins and the anatomically more ubiquitously expressed hnRNP G protein, distinct activities have been reported for members of other families of splicing regulators expressed in different cell types. For example the nPTB protein is a less splicing repressive isoform of PTB, and nPTB expression leads to splicing switches in neural tissue [37]. The TLE4-T exon is also the first testis-specific splicing target pre-mRNA to be identified that is regulated by RBMY and hnRNP G-T.

We identified direct RNA binding sites for Tra2β within the TLE4 pre-mRNA, but found the hnRNP G family proteins each operate as splicing coregulators (this indirect interaction is labelled as X in Figure 10), even though RBMY and hnRNP G-T proteins can bind in and around TLE4-T. In particular hnRNP G-T strongly binds to a GUU-rich sequence within the TLE4 pre-mRNA immediately downstream of the weak 5′ splice site of TLE4-T. This GUU-rich sequence is the first reported hnRNP G-T target RNA binding site, and differs from the RNA target sequences recently identified for both RBMY and hnRNP G [32],[34]. Hence hnRNP G-T is a sequence-specific RNA binding protein distinct from both hnRNP G and RBMY, and although direct RNA binding does not play a role in the TLE4-T splicing reported in this study, direct protein-RNA interactions through such sites elsewhere in the transcriptome might be functionally important. Given that the binding site we have observed for hnRNP G-T would be perfectly positioned to stabilise interactions of early splicing components with this weak TLE4-T 5′ splice site [38], the RRM-independence suggests splicing activation might even occur after the initial steps of exon recognition and during spliceosome assembly [39]. Consistent with this possibility, hnRNP G has been identified as a component of spliceosomes by proteomic studies [34],[40].

The results presented here also suggest an important function of hnRNP G family proteins is to moderate the activity of other splicing factors like Tra2β. The levels of many splicing regulators including Tra2β are known to be tightly controlled by splicing auto-regulatory feedback loops [41]. Through antagonistic protein interactions hnRNP G family proteins may provide additional checks on Tra2β functional concentration (as opposed to overall level) in the nucleus. In this way hnRNP G family proteins may help control the fidelity of the splicing process by preventing the selection of aberrant splice sites like the TLE4-B 3′ splice site which is only weakly selected in the testis despite the high concentration of Tra2β.

While the TLE4-T exon evolved in the primate lineage and so is not shared in the mouse, functional assays carried out using ectopically over-expressed human TLE4 proteins in zebrafish show the peptide encoded by this exon results in enhanced repression of Wnt/β-catenin signalling by the encoded TLE4 protein. Hence although not essential for Wnt/β-catenin signalling *per se*, TLE4-T splicing may play an important role in modulating specific signalling pathways in the human germline. As well as the Wnt/β-catenin pathway, TLE4-repressed target genes are also regulated downstream of the Notch signalling pathway which plays a critical role in germline development in the worm *C. elegans* and may also be important in mammalian spermatogenesis [42]. Our analysis indicates that the physiological TLE4-T splicing pattern is established at several levels, but particularly through the Tra2β, RBMY and hnRNP G-T proteins. Failure of such signalling pathways may thus underlie the germ cell defects in men without RBMY protein and mice haploinsufficient for hnRNP G-T protein.

## Materials and Methods

### Bioinformatic selection of testis-specific exons

Testis-specific exon hunting was carried out by searching against the HOLLYWOOD-RNA Alternative Splicing Database (http://hollywood.mit.edu/). To obtain a collection of cassette exons expressing in testis, keywords “human” for genus species, “internal” for exon position, “skipped” for splicing characterization, and “included in” “testis” were chosen to limit the search. Other settings were default. All human transcripts were downloaded from Ensembl Database (www.ensembl.org) version 38. Alignment of HOLLYWOOD output exons to Ensembl transcripts was performed by the programme water which is a part of EMBOSS package. Parameters used were: gap opening penalty = 10 and gap extending penalty = 0.5 for each extending nucleotide. Other parameters were default. Exons which exactly matched to Ensembl transcripts were removed from the list. A BLAST search was conducted by MEGA BLAST using default parameters. The top 50 EST/cDNA hits of each exon were retrieved. The accession numbers of all retrieved hit entries were used to track the tissue origin from the NCBI database by program, or if inapplicable, manually. For those cDNAs cloned from a pool of several tissues, if testis was one of the library constituents, those cDNAs were postulated as of testis origin. Meanwhile, weak BLAST hits were eliminated according to any of the following criteria: (1) alignment length lies out of the range: query exon length+/−5 bp; (2) identity is less than 95%; (3) aligned region has more than 5 gaps. The exons with all BLAST hits derived from testis were subject to manual check and exons were removed from the list if they were in UTRs or their splicing patterns were ambiguous. Each of the derived exons was assigned a unique serial number which consisted of two numbers linked by dash. For the first number, “1” indicates the exon has only one EST hit and “2” means more than 1 hit. Figure S1 shows the whole flowchart of the data processing, and Table S1 displays the full list of putative testis-specific exons.

### RT–PCR for examining endogenous splicing of TLE4-T exon

Human RNA preparation and reverse transcription were carried out as previously described [18]. The primers used in RT–PCR were specific for TLE4:

1-85-F (sequence 5′-AGAACTGAACGCCATCATTG-3′) and

1-85-R (sequence 5′-TGGAAGATGGGACTGACCTC-3′).

### Activity assay in zebrafish embryos by ectopic overexpression

IMAGE CLONE 5296117 (BC059405) containing a full length cDNA clone of hTLE4 in pBluescript was purchased from Geneservice. In order to obtain the full length cDNA of hTLE4(T), reverse-PCR mediated mutagenesis was carried out using primers

5′-cacacctggatcattaaagCAACAACTCCAGGCCCAGCA-3′ and

5′- tcacttttattctttttctcCCCAATGATGGCGTTCAGTTC-3′

(the uppercase letters correspond to the TLE4 coding sequences, and the lowercase letters correspond to the sequence of the alternative exon TLE4-T). Both constructs were linearized by *Kpn*I and mRNA was synthesized by using the T7 mMACHINE kit (Ambion). 400 ng/µl of each mRNA was micro-injected into 1–2 cell stage zebrafish embryos with a pressure injector. Injection dose was 1 nl per embryo. Injected embryos were collected at the dome stage and RNA *in situ* hybridization was carried out as described [43]. Live embryos were observed at 1 day post-fertilization except indicated otherwise.

### Luciferase assay for Topflash activity

TOPFLASH assay in zebrafish embryos was used to monitor the Wnt/β-catenin signalling activity as described [31]. 100 pg of Topflash construct and 10 pg of Renilla reporter were mixed and injected into 1-cell stage zebrafish embryo, and the manipulated embryos were subsequently injected with indicated RNA. Embryos were allowed to develop until bud stage, then sets of 20 embryos were lysed in passive lysis buffer (Dual-Luciferase Reporter Assay System, Promega) and the luciferase activity were measured with a Berthold luminometer as described [44]. Each sample was analyzed in triplicate, and mean value and standard derivation were calculated. Student t-test was used to evaluate the statistical differences.

### HnRNP G-T constructs

Human hnRNP G and hnRNP G-T were cloned in frame into pGFP3 [18] using standard PCR cloning techniques. The human hnRNP G-T reading frame was amplified by PCR from a PAC template (hPAC292I15) with primers GT_pGEX_Eco (sequence 5′-AAAAAAAAGAATTCATGGTTGAAGCGGATCGCCC-3′) and GT_pGEX_Sal (sequence 5′-AAAAAAAAGTCGACCTTAGTATCTGCTCCGGCCTC-3′).

The PCR product was digested with *Eco*RI and *Sal*I and ligated to the vectors pGFP3 and pGEX-5X1 (GE) previously digested with *Eco*RI and *Xho*I. The hnRNP G-T ΔRNP1 GFP fusion construct versions were made by overlap PCR with primers

GT_delta1_F (sequence 5′-cgagaaaccaacaagagccccgcagacgccaaggc-3′) and

GT_delta1_R (sequence 5′-cgtctgcggggctcttgttggtttctcggtctttc-3′).

### Minigene splicing experiments

Exon TLE4-T together with ∼600 bp of flanking intron sequences were amplified by PCR using BAC RP11-243D20 (purchased from BACPAC Resources Center: http://bacpac.chori.org/) as template. The primers were

1-85-MF (sequence 5′-aaaaaaaacaattggaggcagcagatttacct-3′) and

1-85-MR (sequence 5′-aaaaaaaacaattggacacacagccactttc-3′).

The PCR amplicon was then digested by *Mfe*I and ligated to minigene vector pXJ41 to construct the full length TLE4-T minigene. Ten smaller minigenes were constructed with progressively shorter insertions using the same strategy with the primers:

1-85M-F2 (sequence 5′-aaaaaaaacaattgggatttctgttcccctga-3′);

1-85M-F3 (sequence 5′-aaaaaaaacaattgaccaaacaaggacctcagga-3′);

1-85M-R2 (sequence 5′-aaaaaaaacaattgacgcctgtaatcccaacact-3′);

1-85M-R3 (sequence 5′-aaaaaaaacaattgcagtgagccatgattgtgct-3′);

1-85M-R5 (sequence 5′-aaaaaaaacaattgaaaaaaaaacccaagcctggcgcag-3′).

Cell culture and transfections were as described previously [18]. To enable analysis of the splicing pattern as well as monitoring the expression of transfected splicing factors, each set of transfected cells was split intro two portions: one was subject to RT–PCR; the other portion of cells was lysed in protein sample loading buffer and analyzed by Western blot using anti-GFP, anti-HA, anti-Xpress (Invitrogen) or anti-His tag antibody depending on the expressed protein. Transfections were adjusted so that similar expression levels were detected for Tra2β-GFP, RBMY-GFP, hnRNP G-T-GFP and hnRNP G-GFP and experiments were replicated multiple times. The level of endogenous actin for each sample was monitored as protein loading control. These experiments excluded the trivial explanation that any observed differences in TLE4-T alternative splicing might be the result of differences in expression levels of the various GFP fusion proteins between individual transfections.

The hnRNP A1 knockdown was performed by using siRNA duplex:

sense strand: 5′-CAGCUGAGGAAGCUCUUCA-3′ (Eurogentech) [45]. The HEK293 cells were plated in 6 well plates and cultured to reach around 30% confluency. SiRNA duplex was transfected with Lipofectamine RNAiMAX (Invitrogen) and Opti-MEM (Invitrogen) following manufacturer's instruction. After 48 h incubation, plasmid DNA was transfected into the cells using GeneJammer (Stratagene). Cells were harvested 24 hours after transfection of the plasmid DNA. Efficient knockdown of hnRNP A1 at the protein level was confirmed by Western blot.

### RNA affinity assay and EMSA

The five plasmids containing fragments 1 to 5 (F1–F5) in Figure 7A of the full length minigene were cloned from PCR products using minigene TLE4-T as template. Primers were:

F1_F AAAAAAAAGGTACCGACCAAACAAGGACCTCAGG

F1_R AAAAAAAAGGATCCTGGACCAGAACTTTTCAAAG

F2_F AAAAAAAAGGTACCGAAAAGTTCTGGTCCAATGG

F2_R AAAAAAAAGGATCCGGTGTGTCACTTTTATTCTT

F3_F AAAAAAAAGGTACCAGGAGAAAAAGAATAAAAGT

F3_R AAAAAAAAGGATCCACCTTTAATGATCCAGGTGT

F4_F AAAAAAAAGGTACCAGGTATTGGGCTTTGGCTGA

F4_R AAAAAAAAGGATCCAGTGAAACCCTGTCTCAAAA

F5_F AAAAAAAAGGTACCGAGACAGGGTTTCACTCTGT

F5_R AAAAAAAAGGATCCGCAGTGAGCCATGATTGTGC

PCR products were digested with *Kpn*I and *Bam*HI and then ligated into pBluescript-SK II (-) (Stratagene). The constructs used in Figure 7C for mapping the binding site of hnRNP G-T were cloned by hybridizing synthesized sense and anti-sense oligos. Plasmids were then linearized by *Xba*I and *in vitro* transcribed into radio-labelled RNA with T7 RNA polymerase. F3 was additionally analysed using RNA affinity assay as described previously [18]., In the RNA affinity assays, F3 specific-binding proteins or proteins bands that showed different density in the F3 lane compared to the control lane were excised and sequenced by mass-spectrometry. The bound proteins were also analyzed by Western blots probing with antibodies anti-Sam68 (SC-333), anti-p68 (PAb204), anti-hnRNP H (AM113) polyclonal antibody, anti-hnRNP G antibody [15], anti-TDP43 (gift from Emanuele Buratti), anti-Tra2β [46], anti-hnRNP A1 antibody (4B10), anti-phospho-SR monoclonal (10H3), anti-9G8 monoclonal antibody, and anti-ASF/SF2 polyclonal antibody.

The EMSA was performed using *in vitro* transcribed F1–F5, or ABwt, ABm and ABm1–ABm6 with purified GST tagged RRMs of RBMY, hnRNP G-T and hnRNP G as described [47]. The GST-fusion proteins were expressed and purified by following [32].

### UV crosslinking assay

UV cross-linking assays was performed as described previously [18] with minor modifications. RNAs were transcribed in the presence of [^32^P]-ATP from the pBluescript plasmid containing inserts cloned between the *Kpn* I and *BamH* I sites.

The crosslinking assay was performed in a standard nuclear extract from HEK293-EBNA cells (control), a Tra2β-enriched nuclear extract, or a ASF/SF2-enriched nuclear extract which were prepared respectively from HEK293-EBNA cells transfected with a Flag-Tra2β or a (His)_6_-ASF/SF2 expressing plasmid based on the pTT3 vector [48]. Incubation of the RNA and nuclear extracts in splicing conditions, UV treatment, RNase treatment and sample analysis were carried out according to [18].

## Supporting Information

Figure S1 Flow chart of bioinformatic analysis plus supplementary information. We analysed the splicing of 21 of 102 putative exons in RNA prepared from different human tissues using RT-PCR and identified testis-specific splicing in 4 exons (1–70, 2–23, 2–49 and 1–85 which is TLE4-T) and 1 alternatively spliced exon (2–50) in a testis specific gene. The expected PCR products including the tested exons are indicated by arrows. This high recovery of testis-specific exons from our screen indicates that this bioinformatic approach is a valid method to identify tissue specific splicing events. However it is crucial to use an early version of Ensembl annotation for efficient recovery of alternative exons. It is likely the reason for this is that before the exhaustive sequencing of ESTs, early versions of Ensembl transcripts represent dominant splicing variants of each gene which do not contain tissue-specific exons. If all the ESTs had been annotated, all the candidate alternative exons would be eliminated in this step.
(5.65 MB TIF)

Figure S2 The SR family proteins examined do not activate splicing of TLE4-T. (A) Western blot showing the protein expression levels of each of the epitope-tagged splicing regulators transiently expressed in the HEK293 cells compared with the level of endogenous actin protein detected in the same cell extract. (B) RT-PCR analysis showing splicing pattern of TLE4-T minigene splicing detected in the RNA made from the same cells as analysed for protein content. Co-expression of any of the SR proteins detected as bound to the TLE4-T exon did not activate TLE4-T splicing, and in fact actually repressed splicing of the TLE4-T exon. Expression of SRp30c and SF2/ASF induced splicing of the TLE4-B exon, and co-expression of SC35 and 9G8 induced splicing of further aberrant splice forms which have not been cloned and sequenced. Of two other proteins which bound to TLE4-T in nuclear extracts, hnRNP H slightly enhanced TLE4-T splicing activation (lane 3) while co-expression of p68 (DDX5) had no effect (data not shown). (C) Bar chart showing quantitation of RT-PCR analysis.
(4.61 MB TIF)

Figure S3 Splicing of exon TLE4-T is repressed by the hnRNP A1 protein. SiRNA depletion of hnRNP A1 leads to a weak activation of the TLE4-T exon. Top panel: the levels of hnRNP A1 and actin in cells treated with siRNAs for hnRNP A1 or non-silencing siRNAs were assayed by Western blotting. Middle panel: splicing of exon TLE4-T encoded by the minigene was assayed in the cells depleted or mock depleted for hnRNP A1. Bottom panel: a bar chart shows quantitation of RT-PCR analysis. Although splicing inclusion of the minigene encoded exon TLE4-T was partially enhanced by depletion of hnRNP A1, it was still largely repressed in somatic cells. Hence down regulation of hnRNP A1 is not sufficient by itself to account for the somatic repression of TLE4-T. Consistent with this, in these same cells siRNA depletion of hnRNP A1 did not activate splicing of the TLE4-T exon for the endogenous pre-mRNA encoded by the genomic TLE4 locus (data not shown).
(2.48 MB TIF)

Table S1 Full list of putative testis-specific exons.
(0.04 MB XLS)
