# Supplementary figures and images for: The Germ Cell Nuclear Proteins hnRNP G-T and RBMY Activate a Testis-Specific Exon
Source: PLoS Genet. 2009 Nov 6;5(11):e1000707. doi: 10.1371/journal.pgen.1000707 (PMC2762042; doi:10.1371/journal.pgen.1000707)

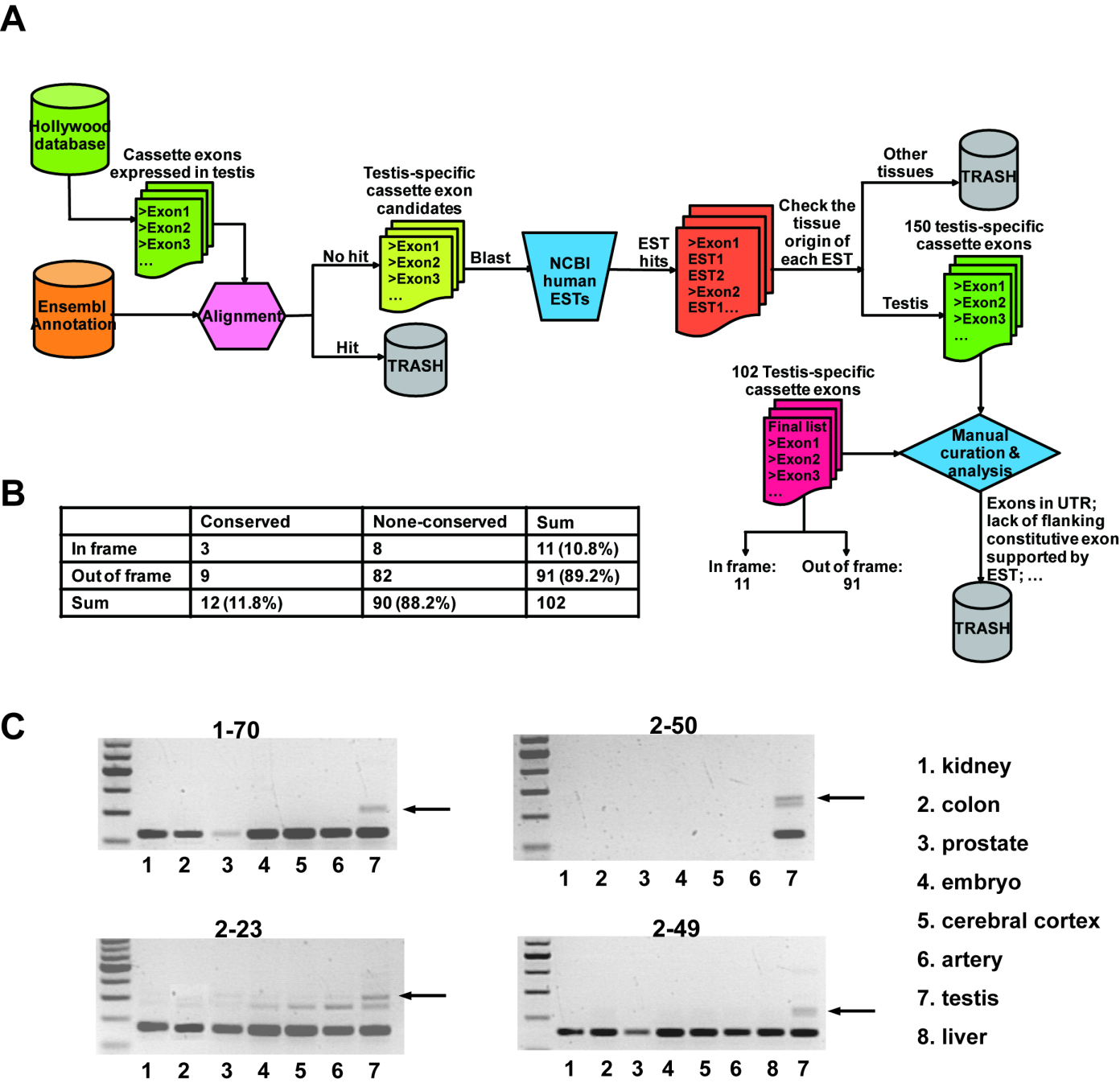

Supplement: Figure S1 — Flow chart of bioinformatic analysis plus supplementary information. We analysed the splicing of 21 of 102 putative exons in RNA prepared from different human tissues using RT-PCR and identified testis-specific splicing in 4 exons (1–70, 2–23, 2–49 and 1–85 which is TLE4-T) and 1 alternatively spliced exon (2–50) in a testis specific gene. The expected PCR products including the tested exons are indicated by arrows. This high recovery of testis-specific exons from our screen indicates that this bioinformatic approach is a valid method to identify tissue specific splicing events. However it is crucial to use an early version of Ensembl annotation for efficient recovery of alternative exons. It is likely the reason for this is that before the exhaustive sequencing of ESTs, early versions of Ensembl transcripts represent dominant splicing variants of each gene which do not contain tissue-specific exons. If all the ESTs had been annotated, all the candidate alternative exons would be eliminated in this step. (5.65 MB TIF) [file pgen.1000707.s001.tif]

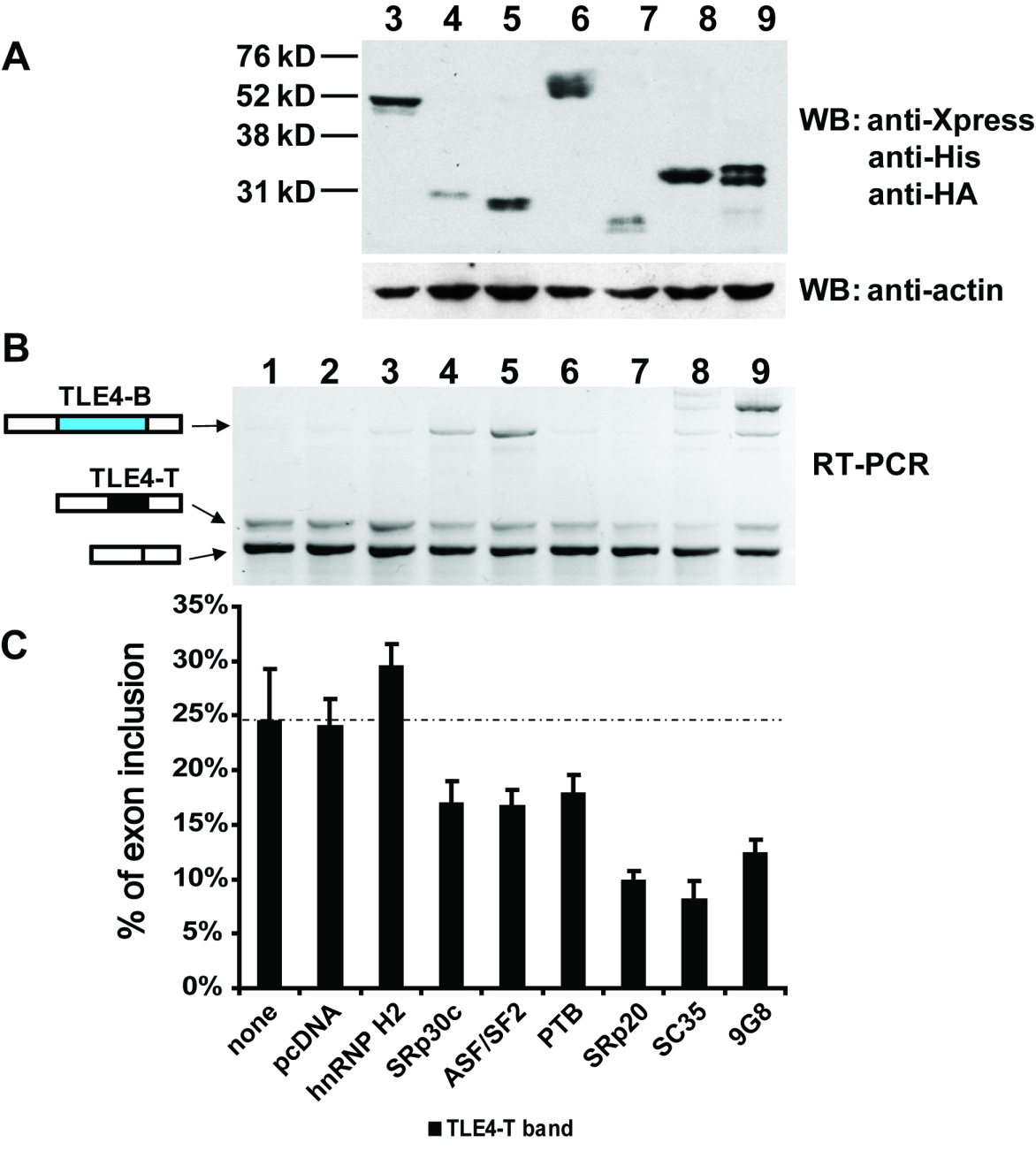

Supplement: Figure S2 — The SR family proteins examined do not activate splicing of TLE4-T. (A) Western blot showing the protein expression levels of each of the epitope-tagged splicing regulators transiently expressed in the HEK293 cells compared with the level of endogenous actin protein detected in the same cell extract. (B) RT-PCR analysis showing splicing pattern of TLE4-T minigene splicing detected in the RNA made from the same cells as analysed for protein content. Co-expression of any of the SR proteins detected as bound to the TLE4-T exon did not activate TLE4-T splicing, and in fact actually repressed splicing of the TLE4-T exon. Expression of SRp30c and SF2/ASF induced splicing of the TLE4-B exon, and co-expression of SC35 and 9G8 induced splicing of further aberrant splice forms which have not been cloned and sequenced. Of two other proteins which bound to TLE4-T in nuclear extracts, hnRNP H slightly enhanced TLE4-T splicing activation (lane 3) while co-expression of p68 (DDX5) had no effect (data not shown). (C) Bar chart showing quantitation of RT-PCR analysis. (4.61 MB TIF) [file pgen.1000707.s002.tif]

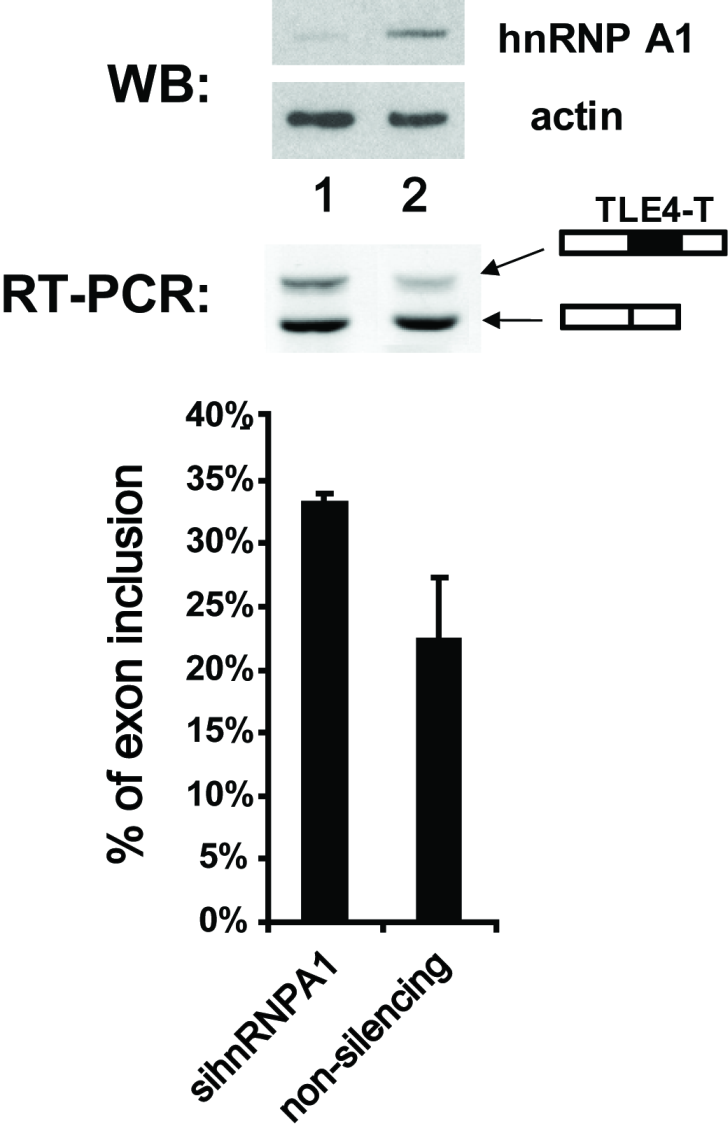

Supplement: Figure S3 — Splicing of exon TLE4-T is repressed by the hnRNP A1 protein. SiRNA depletion of hnRNP A1 leads to a weak activation of the TLE4-T exon. Top panel: the levels of hnRNP A1 and actin in cells treated with siRNAs for hnRNP A1 or non-silencing siRNAs were assayed by Western blotting. Middle panel: splicing of exon TLE4-T encoded by the minigene was assayed in the cells depleted or mock depleted for hnRNP A1. Bottom panel: a bar chart shows quantitation of RT-PCR analysis. Although splicing inclusion of the minigene encoded exon TLE4-T was partially enhanced by depletion of hnRNP A1, it was still largely repressed in somatic cells. Hence down regulation of hnRNP A1 is not sufficient by itself to account for the somatic repression of TLE4-T. Consistent with this, in these same cells siRNA depletion of hnRNP A1 did not activate splicing of the TLE4-T exon for the endogenous pre-mRNA encoded by the genomic TLE4 locus (data not shown). (2.48 MB TIF) [file pgen.1000707.s003.tif]
